# Case Report: Co-existence of a novel *EXOC4‐TRHDE* gene fusion with *PML-RARA* in acute promyelocytic leukemia

**DOI:** 10.3389/fonc.2023.1165819

**Published:** 2023-04-21

**Authors:** Xiaodong Liu, Wanting Li, Jian Xiao, Huixiu Zhong, Kun Yang

**Affiliations:** ^1^ Department of Hematology, Zigong First People’s Hospital, Zigong, China; ^2^ Department of Laboratory Medicine, Zigong First People’s Hospital, Zigong, China

**Keywords:** acute promyelocytic leukemia, *EXOC4‐TRHDE*, additional chromosomal abnormality, all-trans retinoic acid, arsenic trioxide

## Abstract

Acute promyelocytic leukemia (APL) is a type of myeloid leukemia with a specific chromosomal translocation t(15;17)(q22; q12) forming the *PML-RARA* fusion gene. However, approximately one third of newly diagnosed patients with APL have additional chromosomal abnormalities. Here, we report a case of APL with co-existence of a novel translocation t(7;12)(q32;q13) involving an out-of-frame fusion between *EXOC4* and *TRHDE*, together with *PML-RARA*. The patient achieved complete remission after treatment with conventional therapy with all-trans retinoic acid (ATRA) and arsenic trioxide (ATO). Although the causative link between *EXOC4‐TRHDE* and *PML-RARA* has yet to be established, the patient had a good response to therapy, suggesting that the *EXOC4‐TRHDE* fusion does not affect the efficacy of combined treatment with ATRA and ATO.

## Introduction

Acute promyelocytic leukemia (APL) is a specific type of acute myeloid leukemia (AML) characterized by the abnormal accumulation of promyelocytes in the bone marrow and coagulation abnormalities (1). The hallmark of classic APL is the specific chromosomal translocation t(15;17)(q22; q12), leading to the formation of the *PML-RARA* fusion gene. The protein products of this gene fusion lead to cell differentiation arrest and apoptosis deficiency, which is the main molecular mechanism of APL genesis (2). Thanks to the recent standardized clinical use of all-trans retinoic acid (ATRA) and arsenic trioxide (ATO), APL has become an acute leukemia that can be cured without the need for hematopoietic stem cell transplantation (3, 4). However, additional chromosomal abnormalities (ACAs) are present in approximately one third of patients with newly diagnosed APL (5). Although their clinical significance remains elusive, characterization of these ACAs is needed to improve our understanding of the treatment of APL and to predict their response to ATRA/ATO. Here we report a case of *de novo* APL with co-existence of a novel translocation t(7;12)(q32;q13) with *PML-RARA*. The patient achieved complete remission after treatment with conventional therapy with ATRA and ATO. Although the influence of the ACA in this case was unclear, we concluded that it did not affect the efficacy of combined treatment with ATRA and ATO.

## Materials and methods

### Case presentation

**Figure 1:**
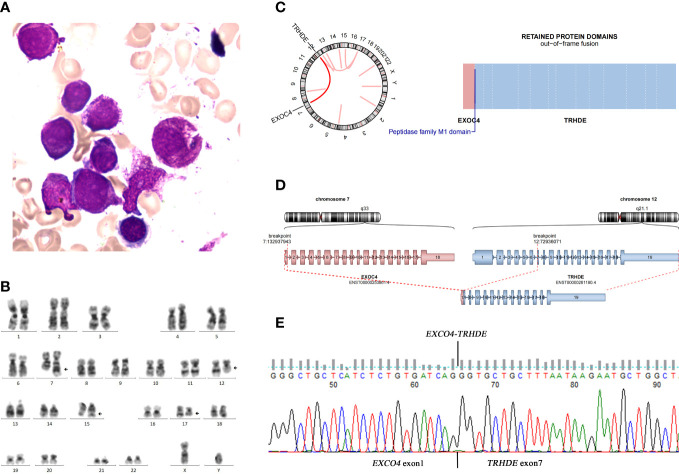
**(A)** Bone marrow aspirate showed hypercellular marrow with increased abnormal promyelocytes. **(B)** Karyotype revealed 46,XY,t(7;12)(q32;q13),t(15;17)(q24;q21)[18]/46,XY[2] in the bone marrow at APL diagnosis. **(C)** Circos plot indicating novel fusions between *EXOC4* and *TRHDE*. **(D)** Total RNA sequencing showed an out-of-frame fusion between exon 1 of *EXOC4* and exon 7 of *TRHDE*. **(E)** Sanger sequencing confirmed the fusion between *EXOC4* and *TRHDE*.

A 59-year-old male with no significant past medical history presented with a 2-day history of fever. The initial blood parameters were as follows: hemoglobin, 7.5 g/dL; white blood cell count, 2.39 × 10^9^/L; neutrophil count 0.7 × 10^9^/L, platelet count, 12 × 10^9^/L, and reticulocytes 1.51%. The prothrombin time and activated partial thromboplastin time were within the normal ranges. Fibrinogen and D-dimer levels were 5.06 g/L and 10.34 mg/L, respectively. A peripheral blood smear showed 55% abnormal promyelocytes. Bone marrow (BM) aspiration revealed hyperplasia with 66% abnormal promyelocytes with numerous cytoplasmic azurophilic granules and Auer rods, including Faggot cells (**Figure 1A**). Cytochemical staining revealed that the abnormal promyelocytes had strong reactivity to myeloperoxidase. Flow cytometric analysis was positive for CD117, CD33, CD13, cMPO, and CD64 (partial), but negative for CD2, CD34, CD79a, human leukocyte antigen (HLA‐DR), CD19, CD20, CD10, CD7, CD3, CD5, CD14, CD36, CD16, cCD3, and CD56. Multiplex real-time polymerase chain reaction showed positivity for *PML/RARA* (bcr-1). Chromosomal analysis revealed 46, XY, t(7;12)(q32;q13), t(15;17)(q24;q21)[18]/46, XY[2] (**Figure 1B**). The molecular features were negative for genetic mutations (*FLT3*, *dupMLL*, *IDH1*, *IDH2*, *NPM1*, *KIT*, *NRAS*, *CEBPA*, *DNMT3A*, *PHF6*, *TET2*, *ASXL1*, *RUNX1*, *TP53*, and *WT1*) associated with AML prognosis at diagnosis.

### Molecular genetics

We characterized the rearrangement involving ACA by total RNA-sequencing of the BM sample from the patient and data were analyzed using STAR software. The Ribozero’s method was utilized to remove ribosomal RNA from total RNA and then to reverse-transcribe it into cDNA to build a library that supports sequencing using cDNA as a template. Whole transcriptome-level detection of RNA from patient samples was carried out *via* the Illumina sequencing platform to analyze transcription-level gene fusions and SNV variants. The sequenced fragments were compared to the UCSC hg19 reference genome using STAR software. Variant detection was analyzed using VarDict software and gene fusion prediction was performed with STAR-Fusion. Along with *PML-RARA*, a novel *EXOC4-TRHDE* fusion, in which exon 1 of *EXOC4* (NM_001037126.1) was fused with exon 7 of *TRHDE* (NM_013381.2), was discovered (**Figures 1C, D**). Sanger sequencing confirmed the fusion between *EXOC4* and *TRHDE* (**Figure 1E**).

### Treatment

The patient was classified as low-risk and received induction chemotherapy with ATRA and ATO. Therapy was complicated by differentiation syndrome and disseminated intravascular coagulation (DIC), which required pausing ATRA therapy and daunorubicin for leukocytosis. BM aspiration following induction therapy showed complete cytologic remission and a normal karyotype. Two weeks after the completion of induction therapy, the patient started consolidation therapy with continuation of ATRA and ATO. The patient tolerated the treatment well, with no major complications. The patient remained in molecular remission for nearly half a year, and BM molecular analysis showed no signs of the fusion transcript.

### Discussion

ACAs are described in all AMLs and occur in approximately one-third of patients with APL (5). The most frequent ACAs in APL are trisomy 8, isoderivative chromosome 17, abnormalities of the long arm of chromosome 7, and trisomy 21 (6). Here we present a case of APL with a novel ACA involving chromosomes 7 and 12 resulting in an out-of-frame fusion between *EXOC4* and *TRHDE*. *EXOC4*, also known as *SEC8*, encodes a subunit in the exocyst complex, which is involved in the tethering of secretory vesicles to the plasma membrane (7). The exocyst complex performs various functions, including, but not limited to, exocytosis, cell growth cytokinesis, and neuronal development (7, 8). Given the essential role of the exocyst complex in cellular and developmental processes, disruption of its functions may be involved in cancer. *EXOC4* has been shown to play a role in a variety of tumors. It was shown to bind directly with c-JNK-interacting protein 4 to regulate mitogen-activated protein kinase signaling cascades in cervical cancer cells (9), modulate transforming growth factor-β-induced epithelial–mesenchymal transition by regulating the expression of N-cadherin and Smad3/4 at the transcriptional level in lung cancer cells (10), and promote metastasis of diffuse-type gastric cancer cells *via* activation of integrin/epidermal growth factor-focal adhesion kinase at Y397 sites signaling (11). Single nucleotide polymorphisms (SNPs) in *EXOC4* might affect TP53 interaction with target gene promoters, ultimately affecting the expression levels of TP53 target genes and clinical outcomes in patients with prostate cancer (12). However, the role of *EXOC4* in hematological tumors remain unclear. Sharda et al. (13) reported that releasates of immature Weibel-Palade bodies from EXOC4-depleted endothelial cells lacked high-molecular weight forms of von Willebrand factor (vWF), demonstrating the importance of EXOC4-mediated endosomal input during vWF maturation. Furthermore, SNPs in *EXOC4* have been associated with impaired platelet aggregation in genome-wide association studies (14), which may be related to the severe DIC in the current patient. Although the mechanism of tumor development promoted by *EXOC4*-*TRHDE* in the presence of *PML-RARA* is unclear, the *EXOC4*-*TRHDE* fusion disappeared during treatment, suggesting that it was sensitive to ATRA and ATO.

Current risk stratification in patients with APL is primarily based on the degree of leukocytosis at diagnosis and influences treatment decisions in clinical practice (1). Although many prognostic variables have been studied to stratify patients with APL, the prognostic relevance of ACAs and complex karyotypes (CKs) remains controversial, and whether the presence of such abnormalities affects treatment decisions in patients with APL is questionable. Opinions on the prognostic influence of ACAs in APL vary. Some studies reported a lack of prognostic impact of ACA in patients with t(15;17) APL treated with ATRA and chemotherapy-based frontline therapies (15–17), while others found that they had a negative impact on outcomes (5, 18). Poire et al. (19) reported that ACAs or CKs were associated with more relapses and significantly poorer survival in patients receiving chemotherapy- or ATO-based consolidation schedules. Additional studies reported that patients with t(15;17) alone were more sensitive to ATRA and had significantly better overall and disease-free survival compared with patients with ACAs, even in the absence of ATO (5, 6). Conversely, however, Labrador et al. (20) and De Botton et al. (21) reported that ACAs did not affect the prognosis of APL patients with t(15;17). Epstein-Peterson et al. (22) demonstrated inferior event-free survival in patients harboring CKs but not in patients with ACAs following frontline ATO-based treatment regimens. Despite the development of severe differentiation syndrome and DIC during treatment, the current patient had a good response to therapy, similar to patients with t(15;17) alone, suggesting that the coexistence of t(7;12)(q32;q13) and bcr-1 isoform did not have any detrimental effect on the response to ATRA and ATO. However, since definitive conclusions cannot be made due to the nature of this case report, the present findings need to be further verified in future studies.

## Conclusion

In the present study, we characterized the t(7;12)(q32;q13) translocation, which created the novel *EXOC4‐TRHDE* fusion gene. To the best of our knowledge, this gene fusion has not previously been described or observed to co-exist with *PML-RARA*. Although the causative link between *EXOC4‐TRHDE* and *PML-RARA* has yet to be established, the patient had a good response to therapy, indicating that the co-existence of t(7;12)(q32;q13) and the bcr-1 isoform did not have a detrimental effect on the response to ATRA and ATO. Further studies are needed to clarify the clinical features and prognosis associated with this ACA.

## Data availability statement

The original contributions presented in the study are included in the article/supplementary material. Further inquiries can be directed to the corresponding author.

## Ethics statement 

The studies involving human participants were reviewed and approved by the Medical Ethics Committee of the First People’s Hospital of Zigong. The patients/participants provided their written informed consent to participate in this study. Written informed consent was obtained from the individual(s) for the publication of any potentially identifiable images or data included in this article.

## Author contributions 

KY and XL designed the study, collected the material, analyzed the data, and wrote the manuscript. KY, XL, WL, JX, and HZ collected the clinical samples and the analyzed data. WL participated in analyzing the data and writing the manuscript. All authors contributed to the article and approved the submitted version.
